# Does a Secure Attachment Style Predict High Psychological Resilience in Patients With Cancer Receiving Chemotherapy?

**DOI:** 10.7759/cureus.10954

**Published:** 2020-10-15

**Authors:** Fatma Basal, Seda Onur, Deniz Yamac, Cengiz Karacin, Guliz Zengin, İrem Bilgetekin, Umut Demirci, Berna Oksuzoglu

**Affiliations:** 1 Medical Oncology, HSU Dr Abdurrahman Yurtaslan Oncology Training and Research Hospital, Ankara, TUR; 2 Psychology, Ankara Ataturk Chest Surgery and Chest Disease Training and Research Hospital, Ankara, TUR; 3 Medical Oncology, Ankara Guven Hospital, Ankara, TUR; 4 Oncology, HSU Dr Abdurrahman Yurtaslan Oncology Training and Research Hospital, Ankara, TUR; 5 Medical Oncology, University of Uskudar, Memorial Ankara Hospital, Ankara, TUR

**Keywords:** attachment style, chemotherapy, resilience, cancer

## Abstract

Introduction

To investigate the level of psychological resilience and the impact of attachment styles on the degree of resilience to distress in patients with cancer receiving chemotherapy.

Methods

Patients with cancer receiving chemotherapy were included in the study. Participants were requested to complete the Relationship Scales Questionnaire (RSQ), Resilience Scale for Adults (RSA), and a personal information form during the data collection phase. One-way analysis of variance (ANOVA) was used to compare that parameter among the attachment styles. Logistic regression analysis was carried out to identify independent factors affecting resilience.

Results

A total of 384 individuals were included in this study (mean age 53.5 ± 12.1, 27.1 % male). The RSQ results showed that the attachment styles of 190 (49.5%) participants were secure, whereas 194 (50.5%) subjects had an insecure attachment. The median RSA score of participants with a secure attachment was significantly higher than that of patients with insecure attachment (133.15 ± 16.6 vs. 127.0 ± 20.0, p=0.001). Patients with the RSA score of >130 were more educated, were in better economic condition, had better perceived social support, and had a higher frequency of secure attachment than those defined as low resilient. Logistic regression analysis revealed that poor and medium perceived social support and insecure attachment style independently predicted low resilience (RSA≤130).

Conclusion

This study demonstrates that the secure attachment style in patients with cancer improves stress resilience as compared to the insecure attachment style. Our findings also show that insufficient perceived social support is likely a negative factor in resilience.

## Introduction

Cancer is a potentially life-threatening disease [1]. In this sense, depression and anxiety are mostly seen in cancer patients during their treatment [2]. Many cancer patients experience long-lasting negative psychological effects and perceive it as a traumatic illness due to its sudden onset and unclear nature after receiving diagnosis [3]. These emotional disorders can play a role in their lower quality of life and treatment compliance [4]. However, the individual level of psychological resilience plays a role in patients' lives and in their struggle with cancer's traumatic effect [5].

The diagnosis of cancer leads to profound emotional and physiological effects on patients [1,6]. Since the treatment process (combinations of chemotherapy, radiotherapy, and surgery) is complex and problematic, patients’ physical health, mental well-being, and relationships with loved ones may be adversely affected [1]. In contrast, as shown in studies, cancer and its treatment may be a chance for personal growth, positive life alterations, upgraded emotional and social well-being as a result of resilience [3].

Psychological resilience is defined as the coping ability/response of an individual in the face of threatening conditions like severe diseases and losses [7]. It is associated with the interactions between individuals' various internal and external characteristics that determine their response to stress and stress-related events [8]. High resilience helps individuals adapt to the new demands of their life [9]. Psychological resilience may be considered a fundamental structure functioning as a resistance element against stressful life events such as cancer [10]. Additionally, reduced emotional distress and more adaptive and flexible behaviors can be seen in subjects with high resilience even when they were exposed to traumatic and negative life events [9]. However, resilience is a dynamic mechanism that can be affected by life changes in life; environmental and individual contextual factors, such as social support, coping, optimism, hope; and attachment styles [3,9].

Attachment is a conception that was established by Bowlby as a bonding process between individuals; it is also a duration that begins at birth and affects the whole life [11-12]. In the sense of attachment theory, if the caregiver of a child is harmonious with the child’s needs, it could be a secure base, so that the child can learn to regulate its feelings and cope with stressful situations in childhood and later in adulthood [13]. Avoidant, anxious, and disorganized attachment styles are due to primary caregivers, respectively, who dismiss their attachment-related memories and feelings, anxiously preoccupy with attachment-related issues, and have unresolved concerning losses and traumas in their attachment history [13].

The attachment system gets activated in stressful times such as a life-threatening medical disease. In this sense, advanced cancer causes the patients to return to their first attachment experience by revealing the problems of addiction and loss of control [6]. The security attachment behaviors provide individuals’ psychological adjustment to a new life after the diagnosis of cancer [12]. It was found that securely attached patients with advanced cancer utilized more positive coping strategies while anxiously attached patients had poorer mental health, in the study that evaluated the role of relationship attachment in psychological adjustment to cancer in patients and caregivers [6,14]. In addition, the attachment insecurities - named attachment anxiety and avoidance in the theory - are related to mental disorders while attachment security contributes to emotional well-being and mental health as shown in the study of Mikulincer et al. [15].

The suggestion, based on evidence, is that attachment style and resilience are connected and the attachment style may affect the individual differences in resilience [12]. Secure attachment is seen as a possible resilience factor that can protect individual well-being in the face of risk and distress. In addition, secure attachment represents the quality of regulation and reduces the strength of emotional responses to health events as a source of resilience [16].

There is a lack of study that evaluates the prediction effect of attachment styles on psychological resilience in patients with cancer. We hypothesized that patients with cancer receiving chemotherapy are in demanding and stressful physical and psychological conditions. Thus, the patients who have a secure attachment style are more resilient individuals than patients who have insecure (anxious, avoidant, and disorganized) attachment styles.

We, therefore, aimed to: (1) reveal the attachment styles of cancer patients receiving chemotherapy; (2) describe the resilience degree (high resilient or low resilient) of those patients due to attachment styles as secure and insecure; (3) compare the demographic features according to attachment styles as well as resilience status; (4) find out the predictor effect of attachment security for high resilience.

## Materials and methods

Participants and study design

Four-hundred forty-one patients with cancer receiving chemotherapy were applied the scales in this cross-sectional study performed in two centers (Dr. A.Y. Ankara Oncology Training and Research Hospital, Ankara Güven Hospital). Patients aged between 18 and 85 years and diagnosed with any stage solid tumor at least three months ago were included. Patients who did not have an adequate level of understanding of the Turkish language and those had frank psychiatric disorders, symptomatic brain metastasis, dementia, disturbing cognitive functions, etc. were excluded from the study. The analysis of 384 patients meeting inclusion criteria was performed. Written informed consent was obtained from all participants. The study was approved by the local ethics committee (numbered 2018-07/74) and was conducted in accordance with the Helsinki declaration.

Participants were requested to complete two inventories: The Relationship Scales Questionnaire (RSQ) and the Resilience Scale for Adults (RSA). They also filled a personal information form (demographics, monthly salary, education, marriage status, whom they grew up with and with whom they were living now, perceived social support with a numbered scale, and subjective confidence in chemotherapy). Patients were grouped according to their economic status into three groups: low, middle, and high. Low income was accepted as monthly income up to, and including, minimum wage; middle income was defined as income between minimum wage and triple the minimum wage; while high income was defined as income over three times the minimum wage. Perceived social support was measured on a scale numbered 0-10 (0: Any social support, 10: Full social support). Poor, medium, and well-perceived social support was accepted as 0-3, 4-6, 7-10 on the scale, respectively. Attachment styles other than secure attachment were defined as an insecure attachment in order to be able to better determine the role of secure attachment in resilience. Additionally, to address resilience for a different perspective, we provided two resilience groups (high resilient: RSA score > 130, low resilient: RSA score ≤ 130) according to the mean score.

Relationship Scales Questionnaire

The Turkish version of the RSQ was used to determine the participants’ attachment style [17]. The RSQ contains 30 short statements drawn from three major studies: the attachment measure by Hazan and Shaver, the relationship questionnaire by Bartholomew and Horowitz, and the adult attachment scale by Collins and Read [13,18-19]. Individuals answer the questionnaire concerning a seven-point scale (1 meaning “does not describe me at all” while 7 means “describes me very well”). The ‘secure’ and ‘avoidant’ attachment patterns are associated with five items each while the ‘anxious’ and ‘disorganized’ attachment patterns are associated with four items each. Scores for each attachment pattern are obtained by calculating the mean score of the four or five items representing each attachment prototype (some statements are scored in reverse).

Resilience Scale for Adults

The RSA is a 33-item scale covering six dimensions, which measure resilience as a form of healthy adaptation and personal competence during exposure to significant adversity, trauma, or stress [20]. The Turkish version of the RSA [21] was utilized to evaluate both the intrapersonal and interpersonal protective factors that promote adaptation to adversity (perception of self, planned future, social competence, structured style, family cohesion, and social recourses) with a total score calculated as a combination of each factor. Scores for responses to items range from one to seven; higher scores reflect higher levels of protective factors of resilience.

Primary outcome

The primary outcome measures of this study were the RSA scores of patients with attachment styles (secure vs. insecure) and factors associated with the resilience of participants, including attachment styles. In addition, the independent predictor effect of attachment security for high resilience was the primary outcome.

Statistical analysis

All statistical analyses were carried out on SPSS v.21 (SPSS Inc., Chicago, IL, USA). The Kolmogorov-Smirnov test was used to determine whether variables were distributed normally. The homogeneity of variances was assessed with the Levene test. Data are presented as mean ± standard deviation (SD) for continuous variables and frequency (percentage) for categorical variables. Categorical variables were compared with the Pearson chi-square test. One-way analysis of variance (ANOVA) was used to compare that parameter among the attachment styles (secure/anxious/avoidant/disorganized). When the overall significance was observed, pairwise post-hoc tests were performed using Tukey’s test. After providing the divided two groups of attachment styles (secure vs. insecure), an independent T-test was performed. Logistic regression analysis was carried out to identify independent factors affecting resilience. A p-value of less than 0.05 was accepted to demonstrate statistical significance.

## Results

A total of 384 individuals were included in this study (mean age 53.5 ± 12.1, 27.1% male). The most seen type of cancer was breast cancer (58.6%) and the following types were gastrointestinal cancers (21.9%) and urogynecological cancers (9.4%). Lung cancer (6.8%), head-neck cancer (2.1%), and other types (1.2%) were also detected. Considering the cancer stage at diagnosis, 26% (n=100) patients were in the metastatic stage at diagnosis. In addition, chemotherapy was for adjuvant, neoadjuvant, and metastatic therapy in 45.1% (n=173), 11.2% (n=43), and 43.7% (n=168) patients, respectively.

The mean RSA score was 130.0 ± 18.6. The attachment style of 190 (49.5%) participants was secure while the avoidant attachment was reported in 153 (39.8%), the anxious attachment was reported in 21 (5.5%), and disorganized attachment was defined in 20 (5.2%) of the participants.

Table 1 provides a comparison of the demographic features of the participants according to their attachment styles. The groups were similar with respect to age, educational status, level of perceived social support, persons living in the same household, and persons with whom they grew up with. On the other hand, gender, marital, and economic status were different significantly between the groups. The mean RSA score of the participants with the secure attachment style was significantly higher than the patients with the insecure attachment style (133.1 ± 16.6 vs. 127.0 ± 20.0, p=0.001).

**Table 1:** Comparison of the demographic features and resilience scores of the subjects with respect to the attachment styles

		Secure attachment (n:190)	Insecure attachment (n:194)	p-value
Age, years		53.6 ± 12.5	53.6 ± 12.2	0.811
Female/male, n (%)		126/64 (66.3/33.7)	154/40 (79.4/20.6)	0.003
Marital status, n (%)				0.041
	Married	143 (75.2)	165 (85)	
	Single	14 (7.3)	10 (5.2)	
	Widow	17 (9)	14 (7.2)	
	Divorced	16 (8.5)	5 (2.6)	
Educational status, n (%)				0.854
	Primary school	74 (39)	83 (42.8)	
	Middle school	25 (13.1)	20 (10.3)	
	High school	35 (18.4)	38 (19.6)	
	University	56 (29.5)	53 (27.3)	
Economic status, n (%)				0.045
	Poor	31 (16.3)	48 (24.7)	
	Medium	150 (79)	141 (72.7)	
	Well	9 (4.7)	5 (2.6)	
Lives with, n (%)				0.079
	Family	150 (79)	164 (84.5)	
	Children	21 (11)	22 (11.3)	
	None	19 (10)	8 (4.2)	
Grew up with, n (%)				0.192
	Family	179 (94.2)	173 (89.2)	
	Mother	8 (4.2)	14 (7.2)	
	Other	3 (1.6)	7 (3.6)	
Perceived social support, n (%)				0.291
	Well	63 (33.1)	73 (37.6)	
	Medium	111 (58.4)	99 (51)	
	Poor	16 (8.5)	22 (11.4)	
Type of chemotherapy, n (%)				0.292
Adjuvant		104 (54.7)	112 (57.7)	
Metastatic		86 (45.3)	82 (42.3)	
Resilience Scale for Adults score n (%)		133.1 ± 16.6	127.0 ± 20.0	0.001

**Figure 1:**
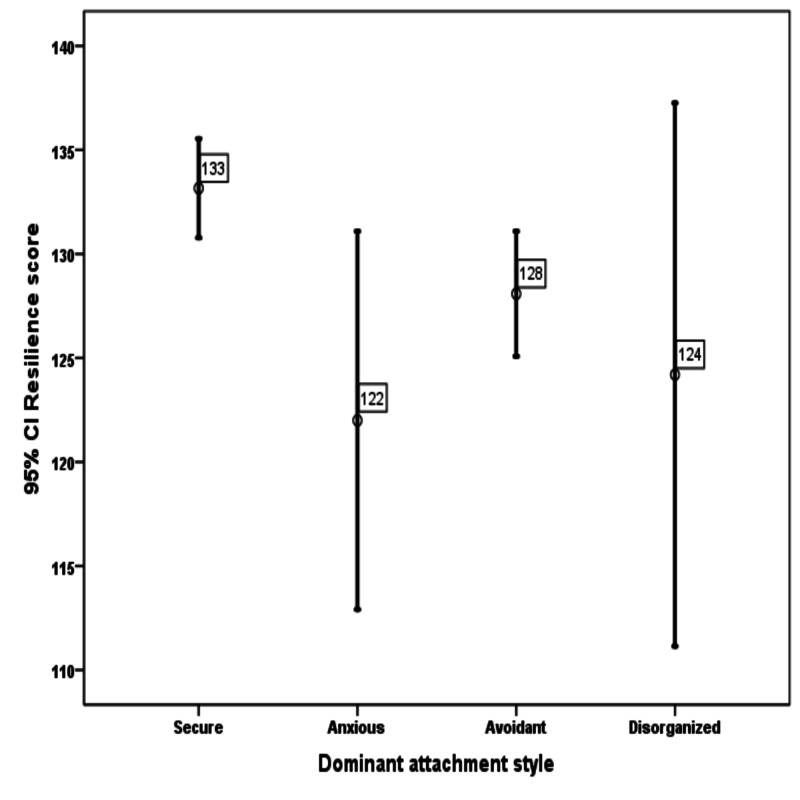
The graphic shows high resilience scores in attachment security as compared to attachment insecurities: anxious, avoidant, disorganized

Considering all the attachment groups separately as secure, anxious, avoidant, and disorganized, individuals were more resilient in the secure attachment style as compared to the other groups (Figure 1). 

A comparison of the demographic features with regard to patients’ resilience is presented in Table 2. The high resilient (RSA score > 130) patients were more educated, were in better economic conditions, and were having better perceived social support as compared to those defined as low resilient (RSA score ≤ 130). Participants in the high resilient group also had a higher frequency of secure attachment than the low resilient group (p=0.007). Consideration in adverse events, including fatigue, vomiting, mucositis, neuropathy, and febrile neutropenia due to chemotherapy were not significantly different in each resilience group (p>0.05).

**Table 2:** Comparison of the patients’ demographic features according to the level of resilience RSA=Resilience Scale for Adults

		Low resilient n:193	High resilient n:191	p-value
Age, years		53.6 ± 12	54.6 ± 12	0.910
Female/ male, n (%)		144/49 (74.6/25.4)	136/55 (71.2/28.8)	0.262
Marital status, n (%)				0.408
	Married	151 (78.2)	157 (82.2)	
	Single	11 (5.7)	13 (6.8)	
	Widow	17 (8.8)	14 (7.3)	
	Divorced	14 (7.3)	7 (3.7)	
Educational status, n (%)				0.010
	Primary school	88 (45.6)	65 (34)	
	Middle school	28 (14.5)	23 (12)	
	High school	39 (20.2)	33 (17.3)	
	University	38 (23.3)	70 (36.7)	
Economic status, n (%)				0.007
	Poor	53 (27.5)	28 (14.6)	
	Medium	135(70)	157 (82.2)	
	Well	5 (2.5)	6 (3.2)	
Lives with, n (%)				0.747
	Family	155 (80.3)	159 (83.2)	
	Children	23 (11.9)	20 (10.5)	
	None	15 (7.8)	12 (6.3)	
Grew up with, n (%)				1.000
	Family	176 (91.2)	174 (91)	
	Mother	12 (6.2)	12 (6.3)	
	Other	5 (2.6)	5 (2.7)	
Perceived social support, n (%)				<0.001
	Poor	27 (14)	11 (5.7)	
	Medium	117 (60.6)	92 (48.2)	
	Well	49 (25.4)	88 (46.1)	
Type of chemotherapy, n (%)				0.436
Adjuvant		107 (55.4)	109 (57)	
Metastatic		86 (45.6)	82 (43)	
Attachment style n (%)				0.007
	Secure	83 (43)	107 (56)	
	Insecure	110 (57)	84 (44)	

Logistic regression analysis to evaluate the independent predictor factors of resilience revealed that poor and medium perceived social support (OR: 3.209, 95% CI: 1.354-7.603, p=0.008 for poor, and OR: 2.129, 95% CI: 1.321-3.430, p=0.002 for medium) and insecure attachment style (OR: 1.751, 95% CI: 1.140-2.692, p=0.011) were independently associated with low resilience (Table 3).

**Table 3:** Independent predictor factors for low resilience

		OR (95% CI)	p
Educational status			
	Primary school graduate	1.00	
	Middle school graduate	0.400 (0.039- 4.082)	0.440
	High school graduate	0.485 (0.049-4.778)	0.535
	University graduate	0.278 (0.028-2.732)	0.272
Economical status			
	Well	1.00	
	Medium	0.717 (0.400-1.287)	0.265
	Poor	1.286 (0.317-5.212)	0.724
Perceived social support			
	Well	1.00	
	Medium	2.129 (1.321-3.430)	0.002
	Poor	3.209 (1.354-7.603)	0.008
Dominant attachment style			
	Secure attachment	1.00	
	Insecure attachment	1.751 (1.140-2.692)	0.011

## Discussion

This study investigated the association between personal attachment style and the degree of resilience to cancer in patients receiving chemotherapy. Our findings show that the patients with a secure attachment style have a higher RSA score indicating a higher degree of resilience to distress. In addition, medium and poor levels of perceived social support, and insecure attachment style are independent predictive factors of low resilience.

Individual attachment is a well-documented indicator of the interpersonal patterns that stem from early childhood interactions with the primary caregiver. The pattern of attachment likely impacts the construction of close relationships, stress resilience, and physiological health [15]. The attachment style of an individual can roughly be categorized as secure or insecure attachment [22]. The secure attachment style is characterized by being comfortable with both intimacy and independence, and resourceful in recruiting necessary help in times of need [23]. The secure attachment style allows a better understanding of oneself and others; thus it improves individuals' resilience in the face of distress [15]. Attachment and stress resilience are considered complementary concepts and share similar developmental circumstances, leading to the emergence of adaptive self-esteem and social empathy. Subjects with secure attachment can maintain their social competence, which allows stress resilience, even under stressful conditions [15]. In this respect, patients with cancer are subject to high levels of psychological stress due to high mortality rates, complex treatment processes, and the complications of chemotherapy [5]. In our study, the high resilience score is found in securely attached patients receiving active chemotherapy.

Resilience is the ability to thrive despite being faced with difficult situations. Provided that the diagnosis of cancer is a critical stress factor for subjects facing it, the resilience of these subjects may influence not only their mental health, well-being, and quality of life but also may impact the progression of cancer [24]. Since resilience is critical in both the mental and somatic health of subjects with cancer, factors influencing resilience need to be clarified. Moreover, several studies have evaluated associations between attachment styles and psychosocial distress in cancer patients [6,14]. Given that attachment style and resilience share similar developmental circumstances and complement each other, we hypothesized that subjects receiving cancer treatment would express a higher degree of stress resilience if they had a secure attachment style. Our findings demonstrate that the secure attachment style is an important component of resilience in patients with a malign solid tumor.

The absence of adequate social support has been shown to increase anxiety and reduce the quality of life in subjects with malignancies [1]. The construction of a strong relationship with family members, spouses, children, and friends can help reduce the stress of dealing with cancer. Having a secure attachment style is associated with being self-sufficient, and such persons may theoretically have better social support, which may influence their resilience when coping with cancer [25]. In contrast to a secure attachment style, subjects with ‘insecure attachment’ may suffer from an impaired psychological adjustment to cancer, which may limit their ability to perceive and access social support [12]. However, according to our findings, no significant difference for perceived social support was detected between attachment styles (secure vs. insecure) while the poor and medium of perceived social support predict low resilience. On the other hand, individuals with an insecure attachment usually have a lower sense of self and a higher sense of others and vice versa, leading to a tendency to overexpress needs. This attachment insecurity causes being generally excessively independent and minimally trusting of others. These individuals with insecure attachment experience difficulties in expressing the distress they are facing and asking for help [26]. In the present study, the perceived social support might be affected by those features of insecure attachment styles that are seen more in the low resilient group than the high resilient group. With a different perspective, the presence of strong social support has also been found to be associated with stress resilience [27]. This finding is also consistent with previous data, which demonstrated the essential role of social support in the development of resilience and the improvement of quality of life in subjects with breast and bladder cancer [27]. On this point, perceived social support and received social support are different from each other, thus it is clear that our limitation is that no social support scale was applied.

In consideration of the demographic features, in contrast to the data provided by De Luca et al., we did not find an association between marital status and the degree of stress resilience [25]. The educational status and economic issues were also associated with resilience in our study population although no predictor effects were revealed. Similar findings were reported in the study of Gao et al., which included Chinese patients diagnosed with oral cancer [28]. The authors stated that higher levels of education, in addition to hope and optimism, were positively and significantly associated with resilience [28]. In the sense of age, different results and evaluations were in studies. Some authors have considered that resilience weakens with older age due to reduced personal resources and physical and cognitive abilities [3]. However, some studies have conceived increased resilience with older age [29]. In the present study, no analysis was performed about older age due to no detection of any differences of age between the high and low resilient groups.

Similar to our study, Schumacher A et al. investigated the resilience in cancer patients undergoing allogeneic stem cell transplantation [3,30]. It was shown that high-resilience patients presented less anxiety and depression, a better quality of life, and higher emotional and social functioning as compared to low resilience patients [30]. In accordance with this study, some studies reported that lower levels of resilience were associated with more distress, depression, cancer-related fatigue, and poorer social adjustment in a long time [3]. In the present study, no differentiation of chemotherapy side effects was found between the low and high resilient groups. However, a lack of assessment of depression, anxiety, psychological well-being reduced our perspectives to resilience and attachment. In this respect, the most deficient aspect of our study is that the scales, including anxiety, depression, well-being, and posttraumatic growth, were not used.

This study has some limitations. Although several factors associated with stress resilience in cancer patients have been identified in this study, the role of these factors on cancer outcome and quality of life have not been investigated. Also, social support was to be evaluated with scale rather than perceived social support. Nevertheless, with the background derived from previous studies reporting significant associations between the level of resilience and quality of life, we speculate that factors influencing resilience may also affect patients’ quality of life and their outcomes. However, further studies are required to address whether factors influencing resilience also contribute to positive outcomes in cancer subjects.

This study investigated the relationship between the personal attachment styles and psychological resilience of patients with cancer receiving chemotherapy for a malign solid tumor. Our findings show that patients with a secure attachment style were high resilient while receiving chemotherapy. Additionally, poor and medium perceived social support and attachment insecurities were negative predictive factors for high resilience. On the other hand, the high resilient patients were more educated and in better economic conditions.

## Conclusions

Our results demonstrate that the secure attachment style in patients with a malignant solid tumor receiving chemotherapy is associated with higher resilience to stress. Providing adequate social support in accordance with the personal attachment style may improve mental and somatic health in subjects with malignancies.
